# Eyelid Laxity, Anthropometric, and Body Composition Parameters: Sex-Based Differences

**DOI:** 10.5152/eurasianjmed.2026.251084

**Published:** 2026-01-13

**Authors:** Thiago Roberto Correia e Silva, Poliana Coelho Cabral, Alcides Da Silva Diniz, Andressa Maranhão de Arruda, Manoela Fernandes Ferreira, Ana Célia Oliveira dos Santos

**Affiliations:** 1 Hospital das Clínicas, Universidade Federal de Pernambuco Faculdade de Ciências Médicas, Empresa Brasileira de Serviços Hospitalares, Recife, Brazil; 2 Departamento de Nutrição, Universidade Federal de Pernambuco, Recife, Brazil; 3 Universidade de Pernambuco Faculdade de Ciências Médicas, Recife, Brazil; 4 Hospital das Clínicas, Universidade Federal de Pernambuco, Empresa Brasileira de Serviços Hospitalares, Recife, Brazil

**Keywords:** Anthropometry, body composition, dermatochalasis, eyelids, skin aging

## Abstract

**Background:**

The physiopathology of eyelid aging (dermatochalasis) involves loss of collagen and elastin. However, few studies have assessed the relationship between anthropometric and body composition parameters and eyelid laxity. This study aimed to explore associations and sex differences between dermatochalasis and such parameters.

**Methods:**

A case series study of 58 patients with dermatochalasis along with a comparison group of 32 normal patients matched by age (50-65 years) and sex was conducted in the ophthalmic clinic of a university hospital. The measures were body mass index, arm circumference (AC), waist circumference, calf circumference (CC), arm muscle circumference (AMC), skin folds, fat mass, grip strength, and change in weight in the previous 5 years.

**Results:**

Men with a lower CC (*P *= .042), AC (*P *= .044), and AMC (*P *= .023) were more likely to have dermatochalasis. A linear regression model revealed that patients with mild and moderate/severe degrees of dermatochalasis tended to have lower AMC (−2.34 cm, *P *= .049) and (−2.80 cm, *P *= .047), respectively. In women, a tendency toward a greater AMC (*P *= .054) was found in those with dermatochalasis, and gains of 5.66 mm in the suprailiac skinfold (*P *= .030) and 1.89 cm in the AMC (*P *= .047) were found in those with mild dermatochalasis.

**Conclusion:**

: Adequate muscle reserve might protect men from eyelid aging, but the same tendency was not confirmed in women. This suggests the use of the diagnosis of dermatochalasis as a proxy variable for the assessment of nutritional disorders in older men.

**Main Points**

- Body composition influences eyelid aging.
- Factors contributing to sagging eyelids.
- Proxy variables for assessing nutritional disorders.
- Sex differences observed in eyelid aging and body composition parameters.
- Lower muscle mass associated with dermatochalasis in men.

## Introduction

### Dermatochalasis

Dermatochalasis, from the Greek *derma *(skin) and *chalasis *(looseness), is the sagging or redundancy of the skin of the upper eyelids,^1^ with the appearance of wrinkles and bags that transform the facial appearance,^2^ especially in middle-aged and older adults.^3^ This upper eyelid aging is a common complaint at ophthalmology offices, especially in oculoplasty.

The physiopathology of the aging of the skin is characterized by the deterioration of structural stability and functional integrity due to accumulated damage resulting from extrinsic and intrinsic factors. A healthy, balanced diet is an important measure for delaying skin aging.^4^^,^^5^

The usual presentation of dermatochalasis is sagging eyelids, typically bilaterally, with functional and aesthetic repercussions, leading to an older appearance incompatible with one’s actual age.^6^ Common complaints include the sensation of weight in the eyelids, irritation due to chronic blepharitis, dry eyes and an undesirable direction of the lashes as well as the obstruction of peripheral vision^7^ or a reduction in vision quality, which eventually negatively impacts daily living activities.^6^

Histological studies have shown that individuals with dermatochalasis have an increase in the number and maximum dilation of lymphatic vessels as well as broad spacing of collagen bundles and the loss of elastic and structural fibers essential to the function of the lymphatic system.^8^ The pathogenesis of dermatochalasis is governed by macrophages and may begin with subclinical inflammation, leading to elastosis and secondary lymphostasis.^8^

Risk factors have been described for sagging eyelids, such as a high body mass index (BMI), advanced age, male sex, light skin, and possibly smoking.^9^ Although prevalent in middle-aged and older individuals and having the proteins collagen and elastin involved in its physiopathogenesis,^10^ no studies have assessed a more comprehensive relationship between anthropometric and body composition parameters and eyelid laxity.

### Body Composition Based on Anthropometrics

Body composition measures provide important information on health and functional capacity. Changes in body composition inherent to the aging process have important consequences for health,^11^ and their effects differ according to the life cycle stage.^12^ With aging, individuals tend to lose muscle mass both quantitatively and qualitatively.^13^

As a direct calculation that involves only weight and height, BMI has the limitation of not providing precise information on body composition. Therefore, different body size measurements and proportions have been used in clinical practice as direct indicators of nutritional status^14^ and skeletal muscle mass, as they can be easily assessed in clinical practice.^15^

The aim of the present study was to examine the associations between upper eyelid aging (dermatochalasis) and alterations in anthropometric and body composition parameters.

## Material and Methods

### Study Design, Setting and Population

Patients with dermatochalasis (58) were enrolled in a case series study with a comparison group of patients without dermatochalasis (32) matched by age and sex. Those patients were under care at an ophthalmology clinic affiliated with a university hospital. This clinic provides care for an average of 600 patients per month.

### Eligibility Criteria

Patients aged 50-65 years of both sexes who were under care at an ophthalmology clinic of a university hospital from August to December 2022 were invited to participate in the study.

Individuals who had undergone eyelid surgery and those with eyelid conditions that could negatively influence the assessment of eyelid flaccidity, such as ptosis, tumors, and inflammatory processes, were excluded. Individuals older than 65 years of age were also not eligible, as dermatochalasis is nearly universal in individuals older than this age.

After clarifications, those who met the eligibility criteria and accepted the invitation signed a statement of informed consent.

### Variables

The dependent variable was sagging eyelids defined on the basis of a 4-level classification scale:^9^ normal (skin of upper eyelid does not touch eyelashes), mild dermatochalasis (skin of upper eyelid touches lashes), moderate dermatochalasis (skin of upper eyelid covers lashes), and severe dermatochalasis (skin of upper eyelid covers eye).

The independent variables were the anthropometric and body composition data obtained during the physical assessment: BMI, arm circumference (AC), waist circumference (WC), arm muscle circumference (AMC), calf circumference (CC), skinfolds, grip strength, fat mass (FM%), lean mass (LM%), and change in weight in the previous 5 years. All measurements were taken following the WHO protocol.^16^

### Data Collection and Analysis

The interviews and assessments were performed by 2 researchers who had undergone training exercises and used calibrated instruments in a specific room for this purpose to ensure greater participant comfort and reduce the occurrence of embarrassment in front of others. Each participant was assessed only in the presence of the researchers and of an accompanier, when present. To ensure the reliability of the clinical findings, the reproducibility of the anthropometric and body composition measures intra- and inter-examiner was tested, and the accuracy was in compliance with the recommended standards.

The BMI was categorized on the basis of WHO recommendations:^16^ underweight (BMI < 18.5 kg/m^2^), eutrophic (18.5 to 24.9 kg/m^2^), overweight (25 to 29.9 kg/m^2^), and obese (≥ 30.0 kg/m^2^).

Tricipital, bicipital, subscapular, and suprailiac skinfolds were measured in a standardized manner, following established protocols.^14^ Arm muscle circumference was measured in centimeters via the formula described by the WHO.^16^ Grip strength, which is a marker of overall body strength, physical fitness, and muscle mass,^14^ was measured as isometric contraction of the hand during the use of a handgrip dynamometer (Saehan^®^, model SH5001) ranging from 0 to 100 kgf and with a precision of 1 kgf.^14^ The participants were also asked about the highest weight they ever had, the highest and lowest weights in the previous 5 years, and their physical type in childhood and adolescence (underweight, overweight, or neither underweight nor overweight).

Body density was calculated via the Durnin and Womersley’s formula.^17^ The result was used to calculate FM% via Siri’s formula.^18^ The LM% was obtained by subtracting FM% from 100%.^14^

For characterization of the study population profile, frequency distribution was used for qualitative variables, and the Kolmogorov–Smirnov test was applied to check the normality of the data. Variables with a normal distribution are expressed as the means and standard deviations, and those with non-normal distribution are expressed as the medians and interquartile ranges. Unpaired Student’s *t*-tests were used for comparisons of means in the analysis of anthropometric and body composition measures according to sex and the occurrence of dermatochalasis.

A linear regression model was run to estimate the differences in anthropometric and body composition measures considering as exposure variables absence *versus* any degree of dermatochalasis (mild or moderate/severe), stratified by sex. Regression coefficients (intercept beta) were estimated, and the corresponding *P* values were calculated. All the statistical analyses were performed in the Stata^®^ program, version 14, and a 5% significance level (*P *< .05) was used. In addition, 95% CIs were calculated.

## Results

Comparability between the case series (58 patients) and the comparison group (32 patients) was statistically homogeneous according to sex and age. Table 1 displays the sample profile. Among the 58 patients with dermatochalasis, 39 (67.2%) had mild dermatochalasis, 17 (29.3%) had moderate dermatochalasis, and 2 (3.5%) had severe dermatochalasis. The mean age was 58.3 years. Women predominated in the sample (71.1%), and most participants had excess weight. According to the BMI classification, 44.5% of the patients were obese, and 32.2% were overweight. When asked how they were considered in childhood and adolescence, 16.9% and 17.8% reported that they were overweight, respectively. The mean highest weight the patients had reached in life was 82.9 kg, and the median age upon reaching this maximum weight was 52 years. The mean highest and lowest weights in the previous 5 years were 78.3 and 67.5 kg, respectively (Table 1).

Table 2 displays the anthropometric characteristics according to the occurrence of dermatochalasis stratified by sex. For analytical purposes, measurements of the left side of the body were used when applicable. Among the men, statistically significant differences were found for CC (*P* = .042), which was lower among those with any degree of dermatochalasis (mild, moderate, or severe), as were AC (*P* = .044) and AMC (*P* = .023). Among women, a tendency toward a difference was observed for AMC, which seemed to be greater among patients with dermatochalasis, although it was not statistically significant (*P* = .054).

Table 3 displays the estimated regression coefficients from the multiple regression related to the differences between the anthropometric and body composition parameters in patients without dermatochalasis (reference) and those with mild and moderate/severe degrees of dermatochalasis, adjusted for sex. Among men, a tendency toward a lower AC was found in patients with mild dermatochalasis (β = −2.77, 95% CI = −6.04; 0.50, *P *= .093) as well as in those with moderate/severe dermatochalasis (β = −3.69, 95% CI = −7.55; 0.18, *P *= .061). Additionally, a tendency toward a lower CC was found in those with moderate/severe dermatochalasis (β = −3.17, 95% CI = −6.74; 0.41, *P *= .080). For AMC, lower means of 2.34 cm and 2.80 cm were found in patients with mild (95% CI = −4.66; −0.01, *P* = .049) and moderate/severe (95% CI = −2.80; −0.04, *P* = .047) dermatochalasis, respectively.

Among women, a gain in anthropometric measures was observed among those with mild dermatochalasis, with increases in suprailiac skinfold (95% CI = 0.58; 10.7, *P* = .030) and AMC (95% CI = 0.03; 3.75, *P* = .047), with greater difference means of 5.66 mm and 1.89 cm, respectively, than in patients without dermatochalasis. Moreover, a tendency toward an increase in the mean difference in AC was detected among women with mild dermatochalasis (β = 2.51, 95% CI = −0.46; 5.48, *P* = .096).

## Discussion

With the changing eras that have brought about advances in health technology, prolonged life expectancy, and changes in lifestyles, the needs of older people have also changed.^19^ Involutional chronic conditions of the eyes have become increasingly relevant in the spectrum of ophthalmological diseases,^20^ including dermatochalasis. The literature is scarce on this condition, which seems to affect 16.3% of individuals (19% of men and 14.4% of women).^9^

The present study revealed, for the first time, an association between lower AMC and the occurrence of dermatochalasis in men. The AMC is a measure used to assess muscle reserve.^14^ This finding is consistent with histological evidence that dermatochalasis occurs in an environment characterized by the loss of protein and muscle mass, which is frequent in the age range of the population studied.

Sex differences are consistently observed in studies of biological aging, regardless of whether the research involves human populations or animal models.^21^ Sex-related variations can stem from a range of factors, including hormonal influences, genetic differences, variations in karyotype, or external factors such as gender-specific behaviors or environmental exposures.^21^Women with mild dermatochalasis had greater AMC. This may be because many of the patients in the study were in the age range compatible with the climacteric period, which has been associated with a reduction in LM%.^22^ However, the nutritional status of women, with a high frequency of excess weight, may suggest a later entrance into menopause,^22^ preserving LM% for a longer period of time than women with lower weight and BMI because of the protective effect of estradiol against sarcopenia.^23^ A limitation of this study is not accounting for women's menstrual status, including whether they are in the climacteric phase, as the menopausal transition itself is associated with significant changes in body composition.^22^

The measurement of AC is a way to assess nutritional status, which indicates the quantity of both LM% and FM%.^24^ A low AC is an indicator of sarcopenia^25^ and malnutrition.^26^

The CC, an easily obtained anthropometric parameter,^27^ is recognized as a marker of muscle mass in older people^16^ and is also considered in the diagnosis of sarcopenia.^28^ The findings revealed, for the first time, an association between a low CC and low AC in men and the occurrence of dermatochalasis.

A correlation between BMI and sagging eyelids has been described.^9^ The results were not in line with this correlation. These findings may be due to differences in population characteristics. Variations in ethnic background, genetic predisposition, cumulative ultraviolet exposure, body fat distribution, and aging patterns across study populations could influence the manifestation of eyelid skin redundancy independent of overall adiposity. Additionally, no correlations were found between eyelid laxity and FM%, LM%, self-reported changes in weight in the previous 5 years, WC, skinfold measurements, or grip strength.

Lower AMC, AC, and CC might increase the risk of sagging eyelids in men; however, the same tendency was not observed in women.

Although limited by the sample size, the present study casts light on nutritional factors associated with dermatochalasis, a prevalent condition among middle-aged and older adults.^3^ The findings suggest a potential sex-specific relationship, particularly in men, between dermatochalasis and nutritional parameters. Due to the cross-sectional design, the associations observed should be interpreted with caution, as they do not imply causality. In this context, dermatochalasis might warrant further investigation in larger, prospective studies, as a simple and fast proxy variable for the assessment of nutritional disorders in this population.

## Figures and Tables

**Table 1.** Sample Profile of Patients 50-65 Years of Age Under Care at the Ophthalmic Clinic of a University Hospital

**Variables**	**Mean, Median or (%)**	**95% CI**
Degree of eyelid laxity^a^ Normal Mild Moderate Severe	32 (35.6) 39 (43.3) 17 (18.9) 2 (2.2)	26.4-45.8 33.6-53.6 12.1-28.2 0.61-7.74
Sex^a^ Female Male	64 (71.1) 26 (28.9)	61.0-79.5 20.5-38.9
Age (years)^b^ Overall sample Women Older women Men Older men	58.3 ± 4.3 55.3 ± 2.8 62.7 ± 1.6 54.9 ± 2.1 62.5 ± 1.9	57.4-59.2 54.7-55.9 62.4-63.0 54.5-55.3 62.1-62.9
Nutritional status (BMI)^a^ Eutrophic Overweight Obesity	21 (23.3) 29 (32.2) 40 (44.5)	15.8-33.0 23.5-42.4 34.6-54.7
How patient was considered in childhood^a^ Underweight Neither underweight nor overweight Overweight	61 (68.5) 13 (14.6) 15 (16.9)	57.6-76.5 8.6-23.2 10.4-25.7
How patient was considered in adolescence^a^ Underweight Neither underweight nor overweight Overweight Highest weight ever in life (kg)^b^ Highest weight in previous 5 years (kg)^b^ Lowest weight in previous 5 years (kg)^b^ Age at which reached highest weight (years)^c^	62 (68.9) 12 (13.3) 16 (17.8) 82.9 ± 20.8 78.3 ± 16.3 67.5 ± 14.9 52 (43-57)	58.7-77.5 7.8-21.9 11.2-26.9 82.5-83.3 74.9-81.7 64.4-70.6 –

Data expressed as ^a^absolute frequency (n) and relative frequency (%). BMI = Body mass index. ^b^mean ± standard deviation and ^c^ median ± interquartile range. Values expressed with 95% CI.

**Table 2.** Anthropometric and Body Composition Measurements According to the Occurrence of Dermatochalasis Stratified by Sex Among Patients 50-65 Years of Age Under Care at the Ophthalmic Clinic of a University Hospital

Anthropometric and Body Composition Measures	Male					Female				
	**Without Dermatochalasis** **(n = 8)**	**95% CI**	**With Dermatochalasis** **(n = 18)**	**95% CI**	***P***	**Without Dermatochalasis** **(n = 24)**	**95% CI**	**With Dermatochalasis** **(n = 40)**	**95% CI**	***P***
Circumference (cm)										
Arm	32.9 ± 4.1	29.5-36.3	29.8 ± 3.1	28.2-31.3	**.044**	31.1 ± 4.9	29.0-33.2	32.8 ± 5.5	31.0-34.5	.192
Waist	105.8 ± 12.9	95.0-116.6	100.9 ± 16.3	92.5-108.7	.463	94.9 ± 15.6	88.3-101.5	99.7 ± 12.6	95.7-103.7	.188
Calf	39.0 ± 4.2	35.5-45.5	36.1 ± 2.6	34.8-37.4	**.042**	35.6 ± 3.3	34.2-36.9	36.6 ± 3.7	35.1-37.5	.274
Skinfolds (cm)										
Tricipital	15.0 ± 8.4	7.9-22.0	13.3 ± 6.4	10.1-16.5	.569	25.2 ± 10.9	20.6-29.8	26.8 ± 8.8	23.9-29.6	.529
Bicipital	10.2 ± 5.7	5.4-19.6	8.9 ± 4.6	6.6-11.2	.541	17.5 ± 8.4	13.9-21.0	18.1 ± 7.8	5.6-10.6	.774
Subscapular	24.5 ± 5.0	20.3-28.7	26.4 ± 11.5	20.7-32.1	.663	27.8 ± 15.6	21.2-34.4	36.6 ± 3.7	35.4-37.8	.502
Suprailiac	21.4 ± 9.9	13.1-29.7	17.6 ± 8.1	13.6-21.6	.316	23.4 ± 10.1	19.2-27.6	27.6 ± 8.5	24.9-30.3	.076
Grip strength (kgf)										
Right	36.9 ± 5.9	31.9-41.8	39.9 ± 6.8	36.5-43.3	.282	21.2 ± 6.8	18.3-24.1	22.6 ± 6.9	20.4-24.8	.443
Left	34.5 ± 6.0	29.5-39.5	36.8 ± 6.6	33.4-40.2	.415	20.1 ± 5.8	17.6-22.5	21.7 ± 6.1	19.7-23.6	.294
Body mass index (kg/m^2^)	31.2 ± 6.1	26.1-36.3	27.6 ± 5.9	24.6-30.5	.178	28.6 ± 6.4	25.9-31.3	30.4 ± 6.1	28.4-32.5	.270
Fat mass (%)	31.2 ± 4.9	27.1-35.3	29.7 ± 6.1	26.6-32.7	.544	40.3 ± 6.7	34.5-43.1	42.2 ± 4.7	40.7-43.7	.227
Lean mass (%)	68.8 ± 4.9	64.7-72.9	70.3 ± 6.4	67.1-73.5	.628	59.7 ± 6.7	56.8-62.5	57.7 ± 4.7	56.2-59.2	.227
Arm muscle circumference (cm)	28.6 ± 2.7	26.3-30.8	26.1 ± 2.3	24.9-27.2	**.023**	23.1 ± 2.1	22.2-23.9	24.8 ± 3.8	23.6-26.0	.054

Unpaired Student’s *t*-test. Bold indicates statistically significant P. Values expressed with 95% CI.

**Table 3.** The Estimated Regression Coefficients from the Multiple Regression Analysis Relating Anthropometric and Body Composition Parameters with Dermatochalasis According to Sex Among Patients Under Care at the Ophthalmic Clinic of a University Hospital

Anthropometric and Body Composition Measures	Male		Female	
	**Mild** **β (95% CI) (** ***P*** **)**	**Moderate/Severe** **β (95% CI) (** ***P*** **)**	**Mild** **β (95% CI) (** ***P*** **)**	**Moderate/Severe** **β (95% CI) (** ***P*** **)**
Circumference (cm)				
Arm	−2.77 (−6.04; 0.50) (0.093)	−3.69 (−7.55; 0.18) (0.061)	2.51 (−0.46; 5.48) (0.096)	0.36 (−3.29; 4.01) (0.844)
Waist	−2.77 (−17.4; 11.8) (0.699)	−9.06 (−26.4; 8.2) (0.290)	5.78 (−2.00; 13.5) (0.143)	2.60 (−6.96; 12.1) (0.589)
Calf	2.71 (−5.73; 0.31) (0.077)	−3.17 (−6.74; 0.41) (0.080)	1.49 (−0.52; 3.49) (0.145)	0.07 (−2.40; 2.54) (0.957)
Skinfolds (cm)				
Tricipital	−1.50 (−8.26; 5.26) (0.651)	−2.17 (−10.2; 5.84) (0.581)	2.16 (−3.27; 7.59) (0.430)	0.36 (−6.30; 7.03) (0.913)
Bicipital	−0.67 (−5.39; 4.05) (0.773)	−2.58 (−8.17; 3.01) (0.349)	0.57 (−3.99; 5.13) (0.802)	0.65 (−4.94; 6.25) (0.816)
Subscapular	2.83 (−6.82; 12.5) (0.550)	0.003 (−11.4; 11.4) (1.000)	4.13 (−3.43; 11.7) (0.279)	−1.33 (−10.6; 7.96) (0.776)
Suprailiac	−3.62 (−11.9; 4.72) (0.378)	−4.04 (−13.9; 5.83) (0.406)	5.66 (0.58; 10.7) (**0.030**)	1.32 (−4.91; 7.55) (0.674)
Grip strength (kgf)				
Right	2.87 (−3.45; 9.20) (0.357)	3.46 (−4.03; 10.9) (0.350)	1.41 (−2.48; 5.31) (0.471)	1.29 (−3.50; 6.08) (0.593)
Left	2.67 (−3.54; 8.88) (0.384)	1.50 (−5.85; 8.85) (0.677)	1.77 (−1.62; 5.16) (0.302)	1.38 (−2.78; 5.54) (0.511)
Fat mass (%)	−0.87 (−6.37; 4.64) (0.748)	−2.79 (−9.31; 3.72) (0.384)	2.55 (−0.56; 5.66) (0.106)	0.61 (−3.21; 4.43) (0.750)
Lean mass (%)	0.87 (−4.64; 6.37) (0.748)	2.79 (−3.72; 9.31) (0.384)	−2.55 (−5.66; 0.56) (0.106)	−0.61 (−4.43; 3.21) (0.750)
Arm muscle circumference (cm)	−2.34 (−4.66; −0.01) (**0.049**)	−2.80 (−5.55; −0.04) (**0.047**)	1.89 (0.03; 3.75) (**0.047**)	1.23 (−1.05; 3.51) (0.287)

Intercept β expressed as difference means between absence *vs* occurrence of dermatochalasis. Bold indicates statistically significant P. Values expressed with 95% CI.

## Data Availability

The data that support the findings of this study are available on request from the corresponding author.
